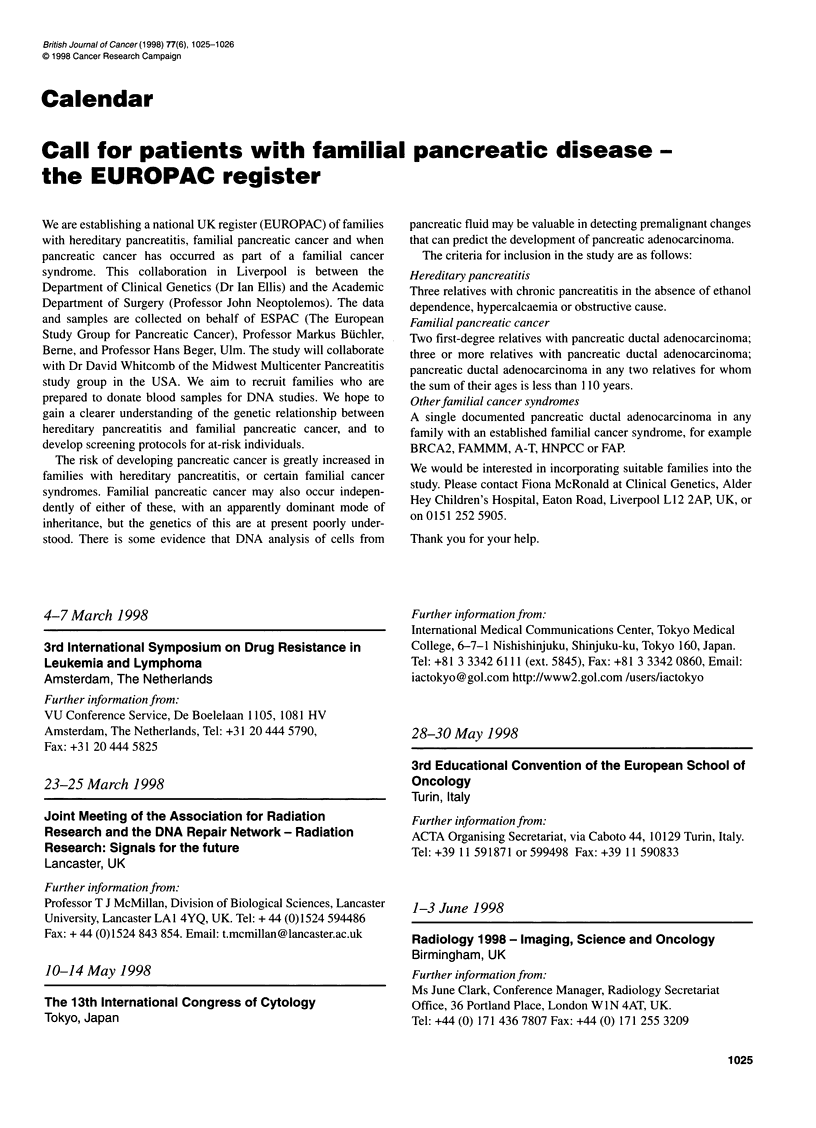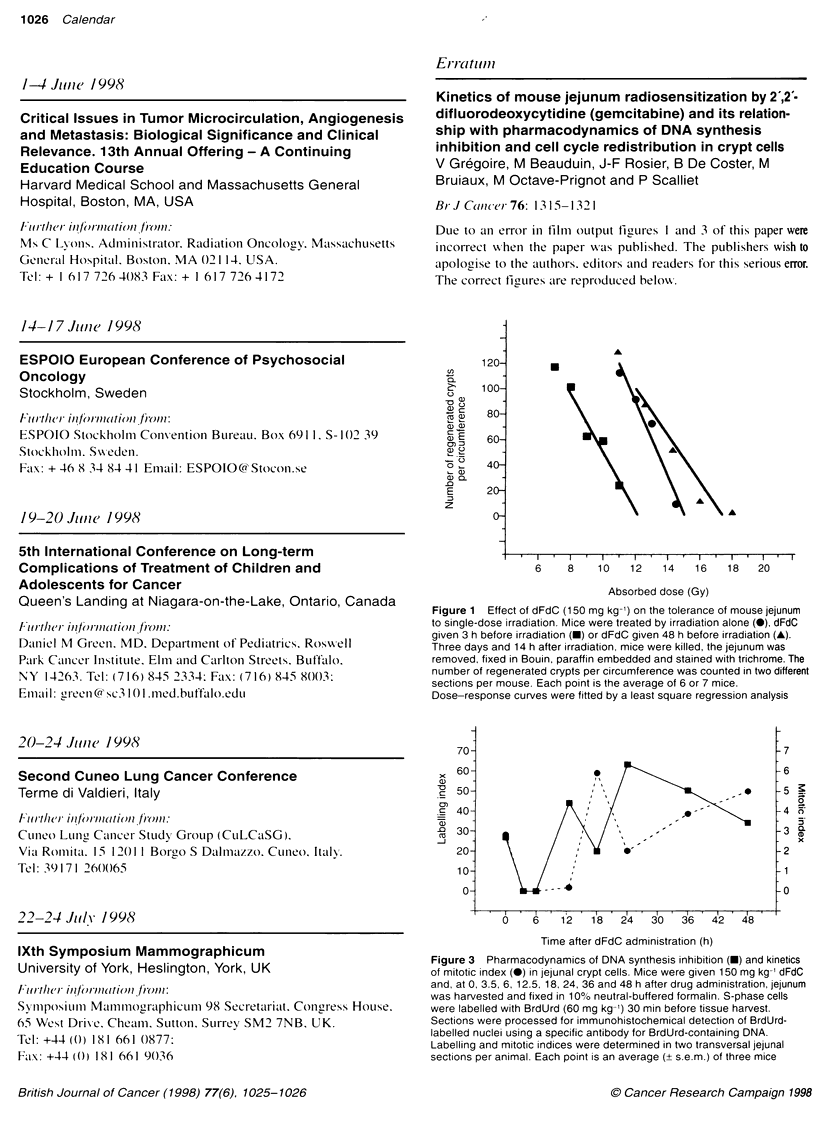# Calendar

**Published:** 1998-03

**Authors:** 


British Journal of Cancer (1998) 77(6), 1025–1026
© 1998 Cancer Research Campaign

### Calendar

### Call for patients with familial pancreatic disease – the EUROPAC register

We are establishing a national UK register (EUROPAC) of families with hereditary pancreatitis, familial pancreatic cancer and when pancreatic cancer has occurred as part of a familial cancer syndrome. This collaboration in Liverpool is between the Department of Clinical Genetics (Dr Ian Ellis) and the Academic Department of Surgery (Professor John Neoptolemos). The data and samples are collected on behalf of ESPAC (The European Study Group for Pancreatic Cancer), Professor Markus Büchler, Berne, and Professor Hans Beger, Ulm. The study will collaborate with Dr David Whitcomb of the Midwest Multicenter Pancreatitis study group in the USA. We aim to recruit families who are prepared to donate blood samples for DNA studies. We hope to gain a clearer understanding of the genetic relationship between hereditary pancreatitis and familial pancreatic cancer, and to develop screening protocols for at-risk individuals.

The risk of developing pancreatic cancer is greatly increased in families with hereditary pancreatitis, or certain familial cancer syndromes. Familial pancreatic cancer may also occur independently of either of these, with an apparently dominant mode of inheritance, but the genetics of this are at present poorly understood. There is some evidence that DNA analysis of cells from pancreatic fluid may be valuable in detecting premalignant changes that can predict the development of pancreatic adenocarcinoma.

The criteria for inclusion in the study are as follows:

*Hereditary pancreatitis*
Three relatives with chronic pancreatitis in the absence of ethanol dependence, hypercalcaemia or obstructive cause.

*Familial pancreatic cancer*
Two first-degree relatives with pancreatic ductal adenocarcinoma; three or more relatives with pancreatic ductal adenocarcinoma; pancreatic ductal adenocarcinoma in any two relatives for whom the sum of their ages is less than 110 years.

*Other familial cancer syndromes*
A single documented pancreatic ductal adenocarcinoma in any family with an established familial cancer syndrome, for example BRCA2, FAMMM, A-T, HNPCC or FAP.

We would be interested in incorporating suitable families into the study. Please contact Fiona McRonald at Clinical Genetics, Alder Hey Children's Hospital, Eaton Road, Liverpool L12 2AP, UK, or on 0151 252 5905.

Thank you for your help.

### *4–7 March 1998*

**3rd International Symposium on Drug Resistance in Leukemia and Lymphoma**
Amsterdam, The Netherlands

*Further information from:*
VU Conference Service, De Boelelaan 1105, 1081 HV Amsterdam, The Netherlands, Tel: +31 20 444 5790, Fax: +31 20 444 5825

### *23–25 March 1998*

**Joint Meeting of the Association for Radiation Research and the DNA Repair Network – Radiation Research: Signals for the future**
Lancaster, UK

*Further information from:*
Professor T J McMillan, Division of Biological Sciences, Lancaster University, Lancaster LA1 4YQ, UK. Tel: + 44 (0)1524 594486 Fax: + 44 (0)1524 843 854. Email: t.mcmillan@lancaster.ac.uk

### *10–14 May 1998*

**The 13th International Congress of Cytology**
Tokyo, Japan

*Further information from:*
International Medical Communications Center, Tokyo Medical College, 6–7–1 Nishishinjuku, Shinjuku-ku, Tokyo 160, Japan. Tel: +81 3 3342 6111 (ext. 5845), Fax: +81 3 3342 0860, Email: iactokyo@gol.com http://www2.gol.com /users/iactokyo

### *28–30 May 1998*

**3rd Educational Convention of the European School of Oncology**
Turin, Italy

*Further information from:*
ACTA Organising Secretariat, via Caboto 44, 10129 Turin, Italy. Tel: +39 11 591871 or 599498 Fax: +39 11 590833

### *1–3 June 1998*

**Radiology 1998 – Imaging, Science and Oncology**
Birmingham, UK

*Further information from:*
Ms June Clark, Conference Manager, Radiology Secretariat Office, 36 Portland Place, London W1N 4AT, UK.
Tel: +44 (0) 171 436 7807 Fax: +44 (0) 171 255 3209

### *1–4 June 1998*

**Critical Issues in Tumor Microcirculation, Angiogenesis and Metastasis: Biological Significance and Clinical Relevance. 13th Annual Offering – A Continuing Education Course**
Harvard Medical School and Massachusetts General Hospital, Boston, MA, USA

*Further information from:*

Ms C Lyons, Administrator, Radiation Oncology, Massachusetts General Hospital, Boston, MA 02114, USA.

Tel: + 1 617 726 4083 Fax: + 1 617 726 4172

### *14–17 June 1998*

**ESPOIO European Conference of Psychosocial Oncology**
Stockholm, Sweden

*Further information from:*

ESPOIO Stockholm Convention Bureau, Box 6911, S-102 39 Stockholm, Sweden.

Fax: + 46 8 34 84 41 Email: ESPOIO@Stocon.se

### *19–20 June 1998*

**5th International Conference on Long-term Complications of Treatment of Children and Adolescents for Cancer**
Queen's Landing at Niagara-on-the-Lake, Ontario, Canada

*Further information from:*

Daniel M Green, MD, Department of Pediatrics, Roswell Park Cancer Institute, Elm and Carlton Streets, Buffalo, NY 14263. Tel: (716) 845 2334; Fax: (716) 845 8003;

Email: green@sc3101.med.buffalo.edu

### *20–24 June 1998*

**Second Cuneo Lung Cancer Conference**
Terme di Valdieri, Italy

*Further information from:*

Cuneo Lung Cancer Study Group (CuLCaSG),

Via Romita, 15 12011 Borgo S Dalmazzo, Cuneo, Italy.

Tel: 39171 260065

### *22–24 July 1998*

**IXth Symposium Mammographicum**
University of York, Heslington, York, UK

*Further information from:*

Symposium Mammographicum 98 Secretariat, Congress House, 65 West Drive, Cheam, Sutton, Surrey SM2 7NB, UK.

Tel: +44 (0) 181 661 0877;

Fax: +44 (0) 181 661 9036